# A pairwise residue contact area-based mean force potential for discrimination of native protein structure

**DOI:** 10.1186/1471-2105-11-16

**Published:** 2010-01-09

**Authors:** Shahriar Arab, Mehdi Sadeghi, Changiz Eslahchi, Hamid Pezeshk, Armita Sheari

**Affiliations:** 1 Department of Bioinformatics, Institute of Biochemistry and Biophysics, University of Tehran, Tehran, Iran; 2 National Institute of Genetic Engineering and Biotechnology, Tehran-Karaj Highway, Tehran, Iran; 3 School of Computer Science, Institute for Research in Fundamental Sciences (IPM), Tehran, Iran; 4 Department of Mathematics and Center of Excellence in Algebraic and Logical Structures in Discrete Mathematics, Shahid Beheshti University, Tehran, Iran; 5 School of Mathematics, Statistics and Computer Sciences, Center of Excellence in Biomathematics, College of Science, University of Tehran, Tehran, Iran

## Abstract

**Background:**

Considering energy function to detect a correct protein fold from incorrect ones is very important for protein structure prediction and protein folding. Knowledge-based mean force potentials are certainly the most popular type of interaction function for protein threading. They are derived from statistical analyses of interacting groups in experimentally determined protein structures. These potentials are developed at the atom or the amino acid level. Based on orientation dependent contact area, a new type of knowledge-based mean force potential has been developed.

**Results:**

We developed a new approach to calculate a knowledge-based potential of mean-force, using pairwise residue contact area. To test the performance of our approach, we performed it on several decoy sets to measure its ability to discriminate native structure from decoys. This potential has been able to distinguish native structures from the decoys in the most cases. Further, the calculated Z-scores were quite high for all protein datasets.

**Conclusions:**

This knowledge-based potential of mean force can be used in protein structure prediction, fold recognition, comparative modelling and molecular recognition. The program is available at http://www.bioinf.cs.ipm.ac.ir/softwares/surfield

## Background

Considering energy function to detect a correct protein fold from incorrect ones is very important for protein structure prediction and protein folding. Mainly, two different types of potential energy function are currently in use, either on the identification of native protein models from a large set of decoys or protein fold recognition and threading studies [1-10]. The first class of potentials, named physical-based potential, is based on the fundamental analysis of the forces between the particles referred to as physical energy function. The second type is knowledge-based energy function based on information from known protein structures. In physical energy function, a molecular mechanics force field is used. Molecular mechanics force fields are parameterized from ab-initio calculation and small molecule structural data. They are essentially the sum of pairwise electrostatic and Van der Waals interaction energies, bonds, angles and dihedral angle terms [11-14]. In addition, terms that are not included such as entropy and the solvent effect are implicitly considered. Although, physical energy function is widely used in molecular dynamic simulation of proteins in their native and denatured states which can be used to distinguish the decoy/native structures, but these functions have not been efficient in protein structure prediction because of their greater computational cost. To reduce the computational complexity of the protein folding problem, knowledge-based or empirical mean-force potential is widely used. Since the structure of folded proteins reflects the free energy of the interaction of all their components, including all enthalpic and entropic contributions, as well as solvent effects, such potentials provide an excellent shortcut towards a powerful objective function. It can be used to force the system to obtain potential between groups of atoms by the use of experimentally determined structures. In this approach, statistical thermodynamics is used in an analysis of the frequency of observed states to estimate the underlying free energy [15]. Most often, the distribution of pairwise distances are used to extract a set of effective potential between residues or atoms. The distribution of pairwise distances can be compiled from the protein structure database and by defining a reference state, Boltzmann equation is used to calculate the interaction energy of a particular pair. The total potential energy of a protein is simply taken as a sum over all pairwise interactions. In most cases, one or two points for each residue are used to represent a protein [16-18]. These points are usually C(alpha), C(beta) or the centre of mass of each side chain. Each interaction can be distance-dependent. A large variety of knowledge-based potential of mean force have been developed by introducing additional interactions such as surface area terms, the main chain and side chain dihedral angles, three and four body terms and heavy atoms [6,19-23].

In the contact potential, either distance-dependent or contact based, the distance between the centres of two C(alpha), C(beta) or centre of mass of two residues or the all heavy atoms of two residues are calculated and the observed frequency of contacts between residues converts to free energy using Boltzmann equation. In this way, two problems may be encountered. First, when an atom or centre of mass is selected for each residue, calculated potential is independent of orientation of the side chains and when the distance between two atoms of two residues are equal to the distance of two atoms of other residues in other positions, the same potentials are assigned to them although the orientation of two residue side chains may be quite different. Second, the atoms of two residues may not have direct contact with each other and some atoms may be located in an interval close to them.

In this study, we develop a new approach to calculate a knowledge-based potential energy using pairwise residue contact area. We calculate the parts of each pairwise residue area that are in contact in Å2 by rolling a probe ball of different sizes around the atoms of a residue to determine the contacts area of each pair. This pairwise contact area is used to determine statistical contact area preference between each residue pairs, when a contact area preference estimates a sum of energetic interactions and structural constraints.

A good energy function at its minimum should discriminate native structures from decoys. So, to test the effectiveness of this new potential, we calculate it on several decoy sets to measure its ability to discriminate native structure from decoys. Several decoy sets that contain one to hundreds of decoy structures generated in different ways are used and in the most cases this potential has been able to distinguish native structures from the decoys. Calculated Z-score and Pe, which are useful measures of the validity of the computed potential, show high value for all protein datasets.

## Results and Discussion

One of the best ways to show the performance of a force field is its ability to find the native structure in a large set of decoys. Different decoys sets have been used to evaluate how well knowledge-based potentials and physical potentials discriminate native structures. In this study, the performance of our model based on pairwise contact area was tested on models from different decoy sets containing *misfold, DecoyForMMPBSA, fisa, hgstructal, semfold, vhp-mcmd, 4state_reduced, lmds, ig_structal, ig_structal_hires, HRDecoy *and *Rosetta_Tsai*. The quality of the models in decoy sets and the members of decoy structures are very different.

From the principle of statistical mechanics, we suppose that the energy of the native structure has to be minimum energy among all conformations and much lower than the average energy of all possible conformations. Then, in addition to finding the fold with lower energy, the Z-Score has been calculated. Energy profiles have been made for each residue pairwise separated by *d *residues in sequence (10 distinct values for sequence separation have been considered) using different probe sizes (*r *= 0.25, 0.5, 0.75, 1, 1.5, 2, 2.5 Å). So, we have 70 different energy profiles for energy calculation. The total energy of particular structure of a protein can be calculated as the sum of all the pairwise interactions, but the best discrimination has been achieved when only energy profile for *d *= 1 has been considered. This shows that contact area of consequent neighbors has more important role in distinguishing native folds from the decoys. In this situation, with *r *= 0.25Å we could discriminate native folds from decoys in almost all decoy sets. The increase in the probe size has had slightly improvement on the discrimination power shown by Z-Score. Since the increase of the probe radius resulted in an increase in the amount of calculations, so choosing the great probe radius was not efficient.

Table 1 shows the results for discrimination of 1417 native folds from more than 1300000 decoys in 12 sets. In the most cases, the native structures have the lowest energy and they have got first rank. The high negative values of Z-Scores show that choosing this energy potential is highly effective [7,24-26].

**Table 1:** Performance of contact area energy for native fold recognition on decoy sets

*Decoy set *	*Number of proteins*	*Average number of decoys per set (~) *	*Contact area energy*		
			
			*Rank 1*	*Z score*	*Pe*
Misfold	23	1	23/23	n/a	n/a
4state_reduced	7	665	7/7	-7.0	-6.5
fisa	4	500	4/4	-0.8	-2
hg_structal	29	29	28/29*	-8.5	-3.4
ig_structal_hires	20	19	20/20	-18.7	-3
ig_structal	61	60	61/61	-31.1	-4.1
lmds	9	450	9/9	-17.0	-6.0
semfold	6	11300	5/6	-13.7	-9.3
vhp_mcmd	1	6255	1/1	-14.7	-8.7
DecoysForMMPBSA	12	30	12/12	-5.9	-3.4
HRDecoy	1215	995	1215/1215	-10.4	-6.9
Rosetta_Tsai	30	1862	0/30*	-0.2	-1.5

• It is noticeable that 1gdm is obsolete and it is replaced by 2gdm in protein data bank.

2gdm is the first in energy ranking.

Tables in Additional file 1 show the details of results for proteins in each decoy set. Although these decoy sets have been produced in different ways andfinding native structures in a set of low quality models would not be difficult, but our pairwise contact area based potential ranked the native structures first in the most cases. There are some exceptions. First, in *semfold*, protein 1nkl has the rank five. Second in *hg_structal*, 1gdm has the rank 17, but in the last version of PDB this protein has been replaced by 2gdm and it is notable that when energy has been calculated for 2gdm, this protein ranked first in decoy structures. In the *Rosetta_Tsai *dataset, our model could not find any native structures in the first rank.

The results obtained on different decoy sets show good performance of our methodology to discriminate native folds. However, an experiment to evaluate the performance of an energy model when performing *ab-inito *folding is to discriminate between the native-like and non-native structures. In Additional file 1, contact area energy is plotted against the RMSD from native structure for native and all decoy structures. Different datasets have different distributions of RMSD for non-native proteins. Usually RMSD's are calculated using C_*α *_distances and residue side chain atoms are not considered. Since our method is based on content area of side chain atoms, then it is very sensitive to orientation of side chains atoms although the change in the position of C_*α *_may not be very large. As shown in plots in Additional file 1, in the most cases decoy structures have RMSD more than 2 Å and in these cases the contact area of side chain atoms may be far from native structure and there is a cosiderable distance between the energy of native and decoy structures in most datasets.

Table 2 shows the comparison of the performances of different methods including *DFIRE *[27], *Rosetta *[28,29], *ModPipe*-*Pair*, *Modpipe*-*surf *[23], *DOPE *[30], *PC2CA *[31], *Force model*, [32], *TE13*, *LHL *[33], and *MJ *[34] together with our model (*surfield*) in recognizing native structures from decoys in three decoy datasets. Our model correctly identifies all 20 native structures in these datasets while other methods do not work well.

**Table 2:** Comparison of results with some other residue-based potential function

Decoy set	Protein	*DFIRE*	*Rosetta*	*ModPipe Pair*	*ModPipe Surf*	*ModPipe Comb*	*Dope*	*PC2CA*	*Force model*	*TE13*	*LHL*	*MJ*	*Surfield*
***4state_reduced ***	1ctf	1	1	1	1	1	1	1	1	1	1	1	1
	1r69	1	2	1	17	1	1	1	8	1	1	1	1
	1sn3	1	1	1	7	1	1	1	23	6	1	2	1
	2cro	1	5	1	103	1	1	1	4	1	1	1	1
	3icb	4	6	15	33	8	1	1	2	-	5	-	1
	4pti	1	1	1	71	1	1	1	13	7	1	3	1
	4rxn	1	1	1	18	1	1	667	85	16	51	1	1

***fisa ***													
	1fc2	254	158	491	1	453	357	1	1	-	-	-	1
	1hdd-c	1	90	293	18	135	1	1	1	-	-	-	1
	2cro	1	26	11	146	19	1	1	1	-	-	-	1
	4icb	1	1	196	2	167	1	1	1	-	-	-	1

***lmds ***													
	1bba	501	174	501	117	444	501	501	1	-	217	-	1
	1fc2	501	291	325	54	222	476	53	1	1	1	1	1
	1ctf	1	1	1	1	1	1	1	1	14	500	501	1
	1dtk	1	9	4	1	1	1	2	1	5	2	13	1
	1igd	1	1	1	3	1	1	1	1	2	9	1	1
	1shf-a	1	5	24	18	7	1	1	1	1	17	11	1
	2cro	1	2	4	28	12	1	1	1	1	1	1	1
	2ovo	1	29	5	8	2	1	1	1	1	3	2	1
	4pti	1	4	1	44	1	1	1	1	-	9	-	1

## Conclusions

The aim of this study was to evaluate a mean force potential based on contact area of residues instead of contact or distance to separate correct from incorrect folds. This was done by calculation of contact area of all atoms of residues considering the Van der Walls spheres of atoms and obtaining a coefficient from training dataset used to quantify pairwise potential in a protein fold. The new potential not only is residue orientation-dependent, but also gives residue contact area in angstrom square for each pair of atoms in adjacent residues that provides a better quantification of atomic interaction than distance-based methods.

The analysis in this work showed that the best definition is the one involving the contact area between Van der Walls spheres of atoms of any two consecutive residues with employing a cut off distance around 0.5 Å. Considering atomic radii, those distances around 5 Å has been considered. This, in fact, is close to the cut off distance considered in the contact-based potential methods. Contact area-based potential was able to recognize the native structures on different decoy sets with a high degree of accuracy, as evident from the Z-score. Only one of 1386 native fold in 11 decoy sets was not ranked first, however this protein, *1nlk *in *semfold *decoy set, was ranked five among 11600 models.

These results show that in addition to the important role of contact area between two atoms in improvement of potential function that reflects the orientation of residue side chain, short range contact between neighbor residues play more important role than long range contact.

## Methods

### Pairwise Contact Area

**Figure 1:**
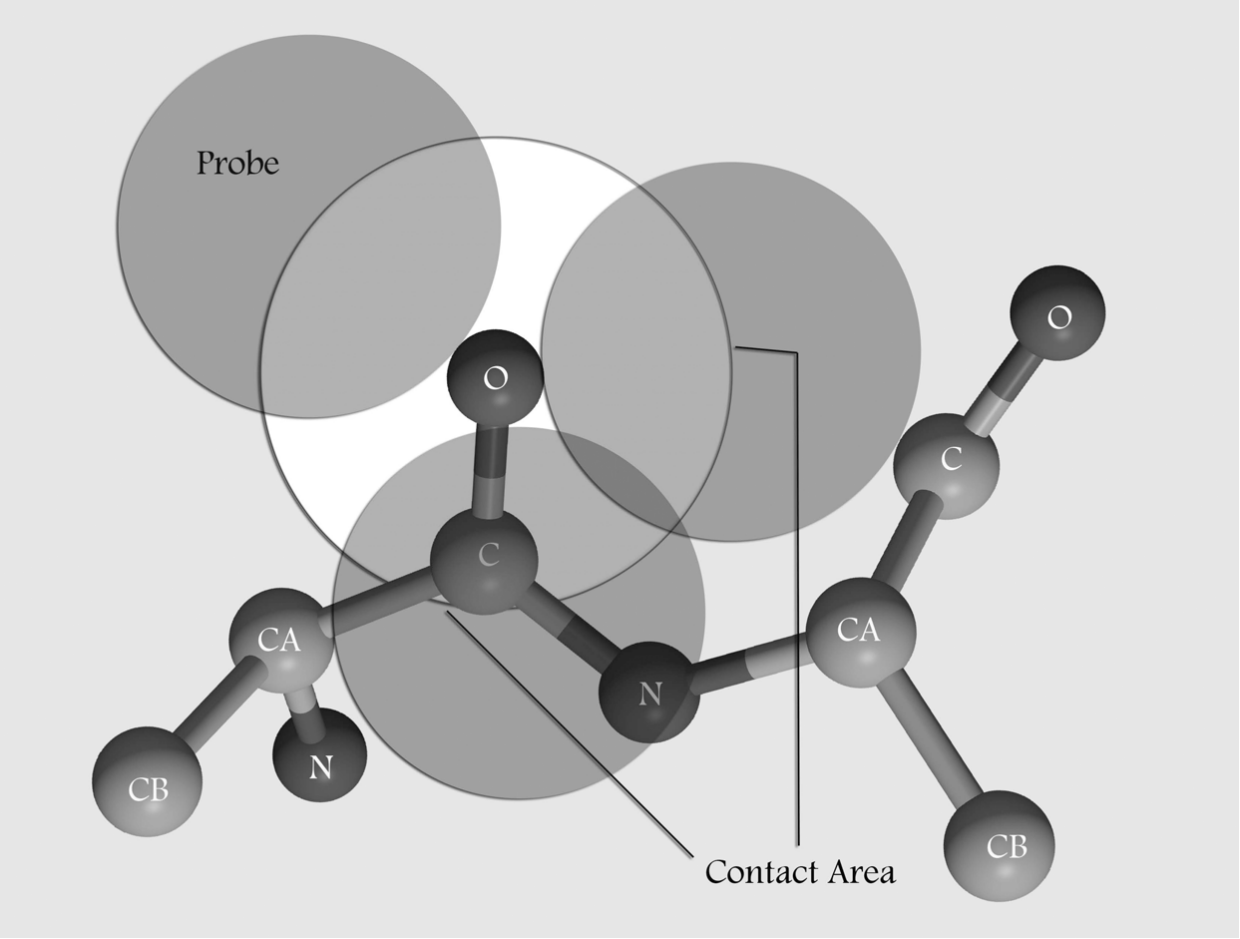
**Contact area of oxygen atom from first amino acid with N, CA and C of next amino acid**.

The presented potential is based on an assessment of contact area for the pairwise residue atoms in a training dataset containing protein structures from protein Data Bank records. The contact area is defined as the faces of sphere of a given atom in a residue contacts to the sphere of an atom in other residue. The radius of the sphere is the atomic Van der Waals radius plus the radius of a probe. The procedure similar to accessible surface area calculation is used to quantify pairwise contact area. For each atom, sufficient number of approximately evenly distribute points are placed on the sphere of radius Ra+Rp centered at the atom where Ra and Rp are the Van der Waals radius of atom A and sphere probe radius respectively (Figure 1). Each point is interpreted as a defined area. In the absence of hydrogen atoms, group radii are used [35]. Table 3 shows the values of atoms radii that are used. Contacting atoms are defined as atoms with overlapping sphere, and thus the maximum distance between two contacting atoms is Ra+Rb+2Rp. In this work, the probe is defined as a sphere with radius 0.25, 0.5, 0.75, 1, 1.5, 2 and 2.5 Angstrom. In theory, by rolling a sphere with radius Rp around an atom, all atoms that have contact with it will be detected and contact area for each residue pair can be calculated. In practice the probe is located at each point on atom sphere and the numbers of points that are in contact with each atom are calculated and the contact area of every two atoms and subsequently for every two residues is calculated.

**Table 3:** Van der Waals radii of atoms

Atom	Radius Å	Atom	Radius Å
C	1.5	F**	2
C**	2	N	1.55
CA	2	ND1	1.55
CB	2	ND2	1.55
CD	2	NE	1.55
CD1	1.75	NE1	1.55
CD1	2	NE2	1.55
CD2	1.75	NH1	1.55
CD2	2	NH2	1.55
CE	2	NZ	1.55
CE1	1.75	O	1.4
CE2	1.75	O**	1.44
CE3	1.75	OD1	1.4
CG	1.75	OD2	1.4
CG	2	OE1	1.4
CG1	2	OE2	1.4
CG2	2	OG	1.4
CH2	1.75	OG1	1.4
CH3	2	OH	1.4
CZ	1.75	S**	2
CZ	2	SD	2
CZ2	1.75	SG	2
CZ3	1.75		

### Training and decoy dataset

A training set containing 562 proteins were obtained from PDB select-25 list [36] by excluding the structures with resolution more than 2.5Å. NMR protein structures and proteins with incomplete side chains or missing atoms were omitted from the training set. All structures were obtained from Protein Data Bank [37]. Proteins presented within any of publicly available decoy sets have been used to test the derived potential function including *DecoysForMMPBSA *[38], *misfold *[39], *fisa *[29], *vhp*_*mcmd *[38], *semfold *[40], *hg*_*structal*, *ig*_*structal*, *ig_structal_hires, lmds *[41], *4state_reduced *[42] (obtained from the decoys 'R' Us web site http://dd.compbio.washington.edu), *HR Decoys*[43] and *Baker's dataset *[44].

### Pairwise Contact Area Potential

The pairwise contact area potential for every residue types *a *and *b *in a given protein are derived from contact area preference within the training set of experimentally determined structures and the contact area of residues type *a *and *b*.

where *E*_(*a*, *b*, *d*, *r*) _is the potential of residue type *a *in contact with residue type *b *separated by *d *residues in sequence calculated using probe with radius *r*. *K(a, b, d, r) *is a coefficient showing the preference of pairwise contact area for a pair of residues (*a*, *b*) in *d *sequence separation by probe radius *r *derived from observed contact area in the training set.

where  is the average of total pairwise contact area of residues(*a*, *b*),  is the average of total pairwise contact area of atom type *a *with all residue types and  is the average of total pairwise contact area of all residue types in *d *sequence separation obtained by probe radius *r*.

These potentials are used to score protein and decoy structures, where the total score is the product of contact area and potential coefficient, summed over all pairwise contact area.

### Measure of significance

#### RMSD

To quantify the similarity of different conformations, we use the coordinate root mean square (cRMS) deviation with the following equation:

where r_ai _and r_bi _are respectively, the *i*_*th *_position of structure *a *and structure *b *when structures *a *and *b *have been optimally superimposed [45].

#### Z-scores and P_e_

The average performance of a potential function to discriminate native structure from decoys can be expressed as Z- score:

where *E*_*native *_is the energy calculated for native protein structure and <*E*_*decoy*_> and *δ *are respectively, the average and the standard deviation of energy distribution of decoy proteins.

A negative Z-score indicates that the conformation's energy is lower than the average of the distribution. The more is the absolute value of the Z-score; the better is the separation of the native conformation from the decoy ones.

Another parameter to compare performance of the various potential functions to discriminate native structures from decoys is based on ranking the decoys by their total potential scores. The parameter is as follows:

where R_native _is the rank of the native structure and N_structures _is the total number of structures in the decoy set. If the rank of the native structure is held constant while the set size is increased, the value of the *P*_*e *_will become more negative (indicating improvement in discrimination capability), while a zero value is the worst possible score indicating the lowest possible rank [46].

## Authors' contributions

All authors participated in the design of the study and interpreting the results. SA implemented the method. The manuscript was written by MS and SA. All authors read and approved the final manuscript.

## Supplementary Material

Additional file 1 **details of results for proteins in each decoy set**. Significance details including Zscore, Pe and scatter plot of energy vs. rmsd for each decoy set are shown in different sheets. Click here for file

## References

[B1] Moult J Comparison of database potentials and molecular mechanics force fields Curr Opin Struct Biol 1997 7 194 199 10.1016/S0959-440X(97)80025-5 9094335

[B2] Vajda S Sippl M Novotny J Empirical potentials and functions for protein folding and binding Curr Opin Struct Biol 1997 7 222 228 10.1016/S0959-440X(97)80029-2 9094333

[B3] Mirny LA Shakhnovich EI How to derive a protein folding potential? A new approach to an old problem J Mol Biol 1996 264 1164 1179 10.1006/jmbi.1996.0704 9000638

[B4] Hao MH Scheraga HA Designing potential energy functions for protein folding Curr Opin Struct Biol 1999 9 184 188 10.1016/S0959-440X(99)80026-8 10322206

[B5] Miyazawa S Jernigan RL An empirical energy potential with a reference state for protein fold and sequence recognition Proteins 1999 36 357 369 10.1002/(SICI)1097-0134(19990815)36:3<357::AID-PROT10>3.0.CO;2-U 10409829

[B6] Lazaridis T Karplus M Effective energy functions for protein structure prediction Curr Opin Struct Biol 2000 10 145 10.1016/S0959-440X(00)00063-4 10753811

[B7] Felts AK Gallicchio E Wallqvist A Levy RM Distinguishing native conformations of proteins from decoys with an effective free energy estimator based on the OPLS all-atom force field and the Surface Generalized Born solvent model Proteins 2002 48 404 422 10.1002/prot.10171 12112706

[B8] Dominy BN Brooks CL Identifying native-like protein structures using physics-based potentials J Comput Chem 2002 23 147 160 10.1002/jcc.10018 11913380

[B9] Lazaridis T Karplus M Discrimination of the native from misfolded protein models with an energy function including implicit solvation J Mol Biol 1999 288 477 487 10.1006/jmbi.1999.2685 10329155

[B10] Jones DT GenTHREADER: an efficient and reliable protein fold recognition method for genomic sequences J Mol Biol 1999 287 797 815 10.1006/jmbi.1999.2583 10191147

[B11] Brooks BR Bruccoleri RE Olafson BD States DJ Swaminathan S Karplus M CHARMM: A program for macromolecular energy, minimization, and dynamics calculations J Comput Chem 1983 4 187 217 10.1002/jcc.540040211

[B12] Lazaridis T Karplus M Effective energy function for proteins in solution. Proteins Proteins 1999 35 133 152 10.1002/(SICI)1097-0134(19990501)35:2<133::AID-PROT1>3.0.CO;2-N 10223287

[B13] Weiner SJ Kollman PA Case DA Singh UC Ghio C Alagona G A new force field for molecular mechanical simulation of nucleic acids and proteins J Am Chem Soc 1984 106 765 787 10.1021/ja00315a051

[B14] Jorgensen WL Maxwell DS Tirado-Rives J Development and testing of the OPLS all-atom force field on conformational energetics and properties of organic liquids J Am Chem Soc 1996 118 11225 111236 10.1021/ja9621760

[B15] Sippl MJ Calculation of conformational ensembles from potentials of mean force. An approach to the knowledge-based prediction of local structures in globular proteins J Mol Biol 1990 213 859 883 10.1016/S0022-2836(05)80269-4 2359125

[B16] Sippl MJ Knowledge-based potentials for proteins Curr Opin Struct Biol 1995 5 229 235 10.1016/0959-440X(95)80081-6 7648326

[B17] Covell DG Folding protein alpha-carbon chains into compact forms by Monte Carlo methods Proteins 1992 14 409 420 10.1002/prot.340140310 1438179

[B18] Sun S Reduced representation model of protein structure prediction: statistical potential and genetic algorithms Protein Sci 1993 2 762 785 10.1002/pro.5560020508 8495198 PMC2142494

[B19] Bauer A Beyer A An improved pair potential to recognize native protein folds Proteins 1994 18 254 261 10.1002/prot.340180306 8202466

[B20] Jernigan RL Bahar I Structure-derived potentials and protein simulations Curr Opin Struct Biol 1996 6 195 209 10.1016/S0959-440X(96)80075-3 8728652

[B21] Melo F Feytmans E Assessing protein structures with a non-local atomic interaction energy J Mol Biol 1998 277 1141 1152 10.1006/jmbi.1998.1665 9571028

[B22] Tobi D Elber R Distance-dependent, pair potential for protein folding: Results from linear optimization Proteins 2000 41 40 46 10.1002/1097-0134(20001001)41:1<40::AID-PROT70>3.0.CO;2-U 10944392

[B23] Melo F Sanchez R Sali A Statistical potentials for fold assessment Protein Sci 2002 11 430 448 10.1110/ps.25502 11790853 PMC2373452

[B24] Dong Q Wang X Lin L Novel knowledge-based mean force potential at the profile level BMC Bioinformatics 2006 7 324 10.1186/1471-2105-7-324 16803615 PMC1534065

[B25] Zhu J Zhu Q Shi Y Liu H How well can we predict native contacts in proteins based on decoy structures and their energies? Proteins 2003 52 598 608 10.1002/prot.10444 12910459

[B26] McConkey BJ Sobolev V Edelman M Discrimination of native protein structures using atom-atom contact scoring Proc Natl Acad Sci U S A 2003 100 3215 3220 10.1073/pnas.0535768100 PMC152272 12631702

[B27] Zhang C Liu S Zhou H Zhou Y An accurate, residue-level, pair potential of mean force for folding and binding based on the distance-scaled, ideal-gas reference state Protein Sci 2004 13 400 411 10.1110/ps.03348304 14739325 PMC2286718

[B28] Misura KM Chivian D Rohl CA Kim DE Baker D Physically realistic homology models built with ROSETTA can be more accurate than their templates Proc Natl Acad Sci 2006 103 5361 5366 10.1073/pnas.0509355103 16567638 PMC1459360

[B29] Simons KT Kooperberg C Huang E Baker D Assembly of protein tertiary structures from fragments with similar local sequences using simulated annealing and Bayesian scoring functions J Mol Biol 1997 268 209 225 10.1006/jmbi.1997.0959 9149153

[B30] Shen MY Sali A Statistical potential for assessment and prediction of protein structures Protein Sci 2006 15 2507 2524 10.1110/ps.062416606 17075131 PMC2242414

[B31] Fogolari F Pieri L Dovier A Bortolussi L Giugliarelli G Corazza A Esposito G Viglino P Scoring predictive models using a reduced representation of proteins: model and energy definition BMC Struct Biol 2007 7 15 10.1186/1472-6807-7-15 17378941 PMC1854906

[B32] Mirzaie M Eslahchi C Pezeshk H Sadeghi M A distance-dependent atomic knowledge-based potential and force for discrimination of native structures from decoys Proteins 2009 77 454 463 10.1002/prot.22457 19452553

[B33] Li X Hu C Liang J Simplicial edge representation of protein structures and alpha contact potential with confidence measure Proteins 2003 53 792 805 10.1002/prot.10442 14635122

[B34] Miyazawa S Jernigan RL Residue-residue potentials with a favorable contact pair term and an unfavorable high packing density term, for simulation and threading J Mol Biol 1996 256 623 644 10.1006/jmbi.1996.0114 8604144

[B35] Pauling L The Nature of the Chemical Bond 1960 3 Ithaca, N.Y.: Cornell University Press

[B36] Hobohm U Sander C Enlarged representative set of protein structures Protein Sci 1994 3 522 524 8019422 10.1002/pro.5560030317 PMC2142698

[B37] Berman HM Westbrook J Feng Z Gilliland G Bhat TN Weissig H Shindyalov IN Bourne PE The Protein Data Bank Nucleic Acids Res 2000 28 235 242 10.1093/nar/28.1.235 10592235 PMC102472

[B38] Fogolari F Tosatto SC Colombo G A decoy set for the thermostable subdomain from chicken villin headpiece, comparison of different free energy estimators BMC Bioinformatics 2005 6 301 10.1186/1471-2105-6-301 16354298 PMC1351271

[B39] Holm L Sander C Evaluation of protein models by atomic solvation preference J Mol Biol 1992 225 93 105 10.1016/0022-2836(92)91028-N 1583696

[B40] Samudrala R Levitt M A comprehensive analysis of 40 blind protein structure predictions BMC Struct Biol 2002 2 3 18 10.1186/1472-6807-2-3 12150712 PMC122083

[B41] Samudrala R Levitt M Decoys'R' Us:a database of incorrect conformation to improve protein structure prediction Protein Sci 2000 9 1399 1401 10.1110/ps.9.7.1399 10933507 PMC2144680

[B42] Park B Levitt M Energy functions that discriminate X-ray and near native folds from well-constructed decoys J Mol Biol 1996 285 367 392 10.1006/jmbi.1996.0256 8627632

[B43] Rajgaria R McAllister SR Floudas CA A novel high resolution Calpha--Calpha distance dependent force field based on a high quality decoy set Proteins 2006 65 726 741 10.1002/prot.21149 16981202

[B44] Tsai J Bonneau R Morozov AV Kuhlman B Rohl CA Baker D An improved protein decoy set for testing energy functions for protein structure prediction Proteins 2003 53 76 87 10.1002/prot.10454 12945051

[B45] Kabsch W A solution for the best rotation to relate two sets of vectors Acta Crystallographica Section D-Biological Crystallography 1976 A32 922 923 10.1107/S0567739476001873

[B46] Reck Gregory M Vaisman Iosif I Decoy Discrimination Using Contact Potentials Based on Delaunay Tessellation of Hydrated Proteins IEEE Computer Society 2007 159 167

